# Scaling Relationships Among the Floral Organs of *Rosa chinensis* var. *minima*: Implications for Reproductive Allocation and Floral Proportionalities

**DOI:** 10.3390/plants14152446

**Published:** 2025-08-07

**Authors:** Zhe Wen, Karl J. Niklas, Yunfeng Yang, Wen Gu, Zhongqin Li, Peijian Shi

**Affiliations:** 1 College of Landscape Architecture, Nanjing Forestry University, # 159 Longpan Road, Nanjing 210037, China; wenzhe@njfu.edu.cn; 2 School of Integrative Plant Science, Cornell University, Ithaca, NY 14853, USA; kjn2@cornell.edu; 3 Bamboo Research Institute, Nanjing Forestry University, # 159 Longpan Road, Nanjing 210037, China; gw0815@njfu.edu.cn (W.G.); zhongqin@njfu.edu.cn (Z.L.)

**Keywords:** allometry, miniature roses, reproductive biomass allocation, scaling analyses, the two-parameter Weibull distribution

## Abstract

Although the allocation of biomass among floral organs reflects critical trade-offs in plant reproductive strategies, the scaling relationships governing biomass allocations remain poorly resolved, particularly in flowers. Here, we report the fresh mass scaling allocation patterns among four floral organs (i.e., sepals, petals, stamens, and carpels), and the two subtending structural components (i.e., the pedicel and receptacle) of 497 flowers of the hypogynous *Rosa chinensis* var. *minima* (miniature rose) using reduced major axis protocols. The two-parameter Weibull probability density function was also applied to characterize the distributions of floral organ mass, and revealed skewed tendencies in all six measured traits. The results show that the numerical values of the scaling exponents (α) for all pairwise power-law relationships significantly exceeded unity (α > 1), indicating disproportionate investments in larger floral structures with increasing overall flower size. Specifically, the scaling exponent of corolla fresh mass vs. calyx fresh mass was α = 1.131 (95% confidence interval [CI]: 1.086, 1.175), indicating that petal investment outpaces sepal investment as flower size increases. Reproductive organs also exhibited significant disproportionate investments (i.e., allometry): the collective carpel (gynoecium) fresh mass scaled allometrically with respect to the collective stamen (androecium) mass (α = 1.062, CI: 1.028, 1.098). Subtending axial structures (pedicel and receptacle) also had hyperallometric patterns, with pedicel mass scaling at α = 1.167 (CI: 1.106, 1.235) with respect to receptacle mass. Likewise, the combined fresh mass of all four foliar homologues (sepals, petals, androecium, and gynoecium) scaled disproportionately with respect to the biomass of the two subtending axial structures (α = 1.169, CI: 1.126, 1.214), indicating a prioritized resource allocation to reproductive and display organs. These findings are in accord with hypotheses positing that floral display traits, such as corolla size, primarily enhance pollen export by attracting pollinators, while maintaining fruit setting success through coordinated investment in gynoecium development. The consistent hyperallometry across all organ pairwise comparisons underscores the role of developmental integration in shaping floral architecture in Rosaceae, as predicted by scaling theory. By integrating morphometric and scaling analyses, this study proposes a tractable methodology for investigating floral resource allocation in monomorphic-flowering species and provides empirical evidence consistent with the adaptive patterns of floral traits within this ecologically and horticulturally significant lineage.

## 1. Introduction

Flowers have played a pivotal role in angiosperm diversification by mediating interactions with pollinators and optimizing reproductive success [1,2]. Perfect flowers (bearing both an androecium and a gynoecium) dominate angiosperm diversity, largely due to their capacity to optimize mating success through pollinator-mediated sexual selection. In contrast, incomplete flowers (e.g., unisexual or structurally specialized forms) often arise from adaptive differentiation to minimize mating costs (e.g., geitonogamy) or to optimize interactions with specific pollinator guilds, such as attracting distinct pollinators or enhancing pollen transfer efficiency, rather than merely reflecting passive adaptations to abiotic factors like wind pollination or resource allocation [3,4]. The spatial and temporal organization of floral organs, including dichogamy (temporal separation of male and female functions, e.g., protandry or protogyny) and herkogamy (spatial separation of anthers and stigmas), serves as a widespread mechanism to minimize self-pollination and optimize outcrossing efficiency across angiosperms, as documented in numerous taxonomic lineages [5,6]. Although textbook examples abound, recent studies highlight context-dependent functional nuances. For example, in protandrous species such as *Rosa* spp., pollen is released before stigmas become receptive, effectively reducing interference between male and female functions [7]. Recent studies further highlight the context-dependent roles of these traits. In duodichogamous chestnuts (*Castanea* spp.), a unique male–female–male flowering sequence enhances female mating success by attracting pollinators via a secondary staminate phase while spatially coupling female flowers with minor pollen emission structures, thereby balancing pollen deposition and self-pollination avoidance [8]. Conversely, in self-incompatible *Epimedium* species, strong herkogamy (spatial separation) proves more effective than partial dichogamy in preventing autonomous and facilitated self-pollination, as weak spatial overlap between anthers and stigmas directly reduces pollen–stigma contact [9]. These findings collectively emphasize that floral trait evolution is shaped by both temporal and spatial strategies, with their relative importance varying across taxa and pollination environments.

Concurrently, corolla and calyx morphology and size directly influence pollinator attraction and handling efficiency. Larger and more symmetrical petals enhance floral visibility and nectar accessibility, as demonstrated across diverse taxa (e.g., *Epimedium* spp. and *Ipomopsis aggregata*) [9,10]. The role of symmetry in pollinator attraction and sexual differentiation is well-documented [11,12,13], underscoring its significance in optimizing male function and outcrossing efficiency. Similarly, calyx integrity protects developing reproductive organs from environmental stressors, a trait critical for maintaining seed viability in variable climates [14]. Such traits are under strong selection, as evidenced by the evolutionary convergence of floral displays in response to pollinator guilds [2,15,16].

**Figure 1:**
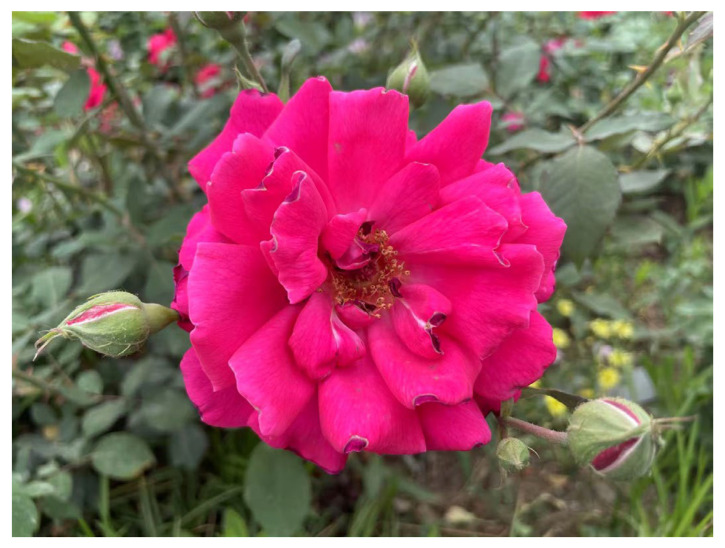
An example of a *Rosa chinensis* var. *minima* flower sampled from a monoculture-dominated urban green space in Nanjing, China (118°55′26″ E, 32°06′00″ N), on 22 April 2025.

Prior research has shown that many organismal traits manifest allometric relationships (i.e., relationships in which two variables fail to scale one-to-one with respect to one another) that can be described by a power-law equation, $Y_{2}=\beta Y_{1}^{\alpha }$ where α is the scaling exponent of two interdependent variables (i.e., *Y*_1_ and *Y*_2_), and β is the normalization constant [17]. Scaling allocation patterns describe how organisms partition resources among competing structures (e.g., floral organs) as overall size changes, revealing trade-offs between growth, reproduction, and maintenance. In botany, scaling analyses have focused generally on vegetative structures, such as leaf biomass–area relationships [18], while floral scaling relationships remain largely underexplored. However, Niklas [3] reported on interspecific floral scaling relationships and showed that across species, gynoecium biomass scales hypoallometrically (α < 1) with perianth mass in perfect flowers, reflecting reduced proportional investment in “female” structures as flower size increases. Herrera [14] documented strong phenotypic correlations between floral organs (e.g., calyx and corolla sizes) in Iberian Papilionoideae (Fabaceae), with calyx–corolla scaling relationships often manifesting isometry (α ≈ 1), whereas fruit morphology exhibited genus-specific diversification uncoupled from floral traits. These data indicate that floral organs may evolve under shared developmental constraints, whereas fruit traits are influenced by divergent ecological pressures, many of which are associated with seed dispersal. However, large-scale analyses of intraspecific floral scaling allocation patterns, particularly among the corolla, reproductive organs, and subtending axial structures, which refers to the pedicel (flower stalk) and receptacle (apical extension of the floral axis), both developmentally derived from stem tissues, are scarce, limiting our understanding of how resource allocation strategies, such as male-biased investment in showy flowers [19], arise from biomass allocation constraints. Addressing this research agenda requires robust datasets from species with measurable floral variation, a criterion met by *Rosa chinensis* var. *minima* (Sims) Voss, whose monomorphic flowers (Figure 1) and horticultural abundance facilitate high-precision measurements, enabling statistically rigorous tests of scaling hypotheses across hundreds of individuals, thereby evaluating how floral biomass partitioning aligns with adaptive strategies in a horticulturally significant system.

The Rosaceae family comprises over 3000 species, many of which are hypogynous, within 100 genera holds immense ecological and economic importance, ranging from wild progenitors (e.g., *Rosa gallica* L. and *R. chinensis* var. *spontanea* (Rehder & E.H. Wilson) T.T. Yu & T.C. Ku) to cultivated hybrids central to global floriculture [20]. Within this family, *R. chinensis* Jacq. (Chinese rose) has played a pivotal role in horticultural history, with its domestication and directed selection reflecting long-term phenotypic modifications [21,22,23,24]. The miniature rose (*R. chinensis* var. *minima*) is a compact hypogynous cultivar bred for urban landscaping, and offers unique advantages for floral scaling analyses. Its small stature simplifies whole-flower sampling, whereas its relatively large floral organs (compared to wild congeners) minimize measurement error when sampled in sufficient quantities. Likewise, its hypogynous flowers permit the careful dissection of carpels (stigmas, styles, and ovaries) to the exclusion of receptacle tissues. Moreover, monoculture plantings, as observed in Nanjing’s urban green spaces, provide homogeneous environmental conditions, reducing ambient environmental “noise” in scaling analyses. However, it is important to note that this sampling design may not capture potential variability associated with ecological or genetic differentiation across natural populations growing in diverse habitats. By leveraging these traits, the goal of this study was to resolve long-standing questions about floral integration and reproductive allocation in a horticulturally and ecologically significant lineage (*Rosa* spp.) using a locally sampled cultivar to bridge gaps between functional morphology and pollination ecology.

## 2. Materials and Methods

### 2.1. Plant Material and Measurements

A total of 497 fresh flowers of *Rosa chinensis* var. *minima* were sampled from a monoculture-dominated urban green space near Xianlin Center Metro Station, Qixia District, Nanjing, China (118°55′26″ E, 32°06′00″ N), on 22 April 2025. Flowers were transported to the laboratory within 30 min for immediate measurement. The fresh biomass of four floral components, i.e., the corolla (petals), calyx (sepals), androecium (stamens), and gynoecium (carpels), and two subtending structures, i.e., the pedicel and receptacle, was measured to the nearest 0.0001 g using an electronic balance (LC-FA1204, Yinzhou Huafeng Electronic Instrument Factory, Ningbo, China). Dry mass measurements were excluded because the small size of stamens and carpels increased the risk of carbonization during drying, leading to unreliable data. Furthermore, prior scaling studies on leaves have demonstrated that fresh mass measurements, when promptly recorded, yield stronger scaling relationships compared to dry mass [25,26].

It is important to note that, although the flowers of *R. chinensis* var. *minima* are described as being hypogynous, some flowers are perigynous (and some are epigynous) owing to developmental variations even on the same plant. Consequently, each flower was carefully dissected to determine the position of the gynoecium and remove carpellary tissues, while also distinguishing and separating these tissues and structures from surrounding receptacles. This process ensured that the gynoecium was accurately isolated from each flower. Therefore, the variation in gynoecium position (hypogynous, epigynous, or perigynous) did not affect the integrity of the gynoecium-specific data collected.

### 2.2. Statistical Analysis of Distribution Models

The frequency distributions of fresh biomass for each floral component were modeled using the normal, log-normal, and two-parameter Weibull distributions. The Weibull probability density function takes the form [27]:

(1) $$f\left(x\right)=\frac{k}{\lambda }{\left(\frac{x}{\lambda }\right)}^{k-1}exp\left[-{\left(\frac{x}{\lambda }\right)}^{k}\right],$$

where *k* (shape parameter) and *λ* (scale parameter) were estimated using maximum likelihood estimation (MLE), which was carried out by the “mle2” function in the package bbmle (v1.0.25.1) based on R (v4.3.1) [28]. The numerical value of the parameter *k* can be used to determine whether the distribution is left-skewed, right-skewed, or symmetrical, wherein the skewness (*S*) of the two-parameter Weibull distribution takes the form:

(2) $$S=\frac{\Gamma \left(1+\frac{3}{k}\right)-3\Gamma \left(1+\frac{1}{k}\right)\Gamma \left(1+\frac{2}{k}\right)+2{\left[\Gamma \left(1+\frac{1}{k}\right)\right]}^{3}}{{\left\{\Gamma \left(1+\frac{2}{k}\right)-{\left[\Gamma \left(1+\frac{1}{k}\right)\right]}^{2}\right\}}^{3/2}}.$$

When *k* < 3.6, *S* > 0, a right-skewed distribution is indicated; when *k* > 3.6, *S* < 0, a left-skewed distribution is indicated; and when *k* = 3.6, *S* = 0, a symmetrical distribution is indicated [29].

Two statistical tests were used to evaluate the suitability of different distribution models for floral organ biomasses: (i) the Shapiro–Wilk test was used to assess normality and log-normality by testing raw and log-transformed data, respectively, and (ii) the Kolmogorov–Smirnov (KS) test was used to evaluate the goodness of fit of the two-parameter Weibull probability density function by comparing empirical data to theoretical quantiles.

### 2.3. Scaling Analysis

Scaling relationships between pairs of floral components were analyzed using the power-law equation (PLE) [17]:

(3) $$Y_{2}=\beta Y_{1}^{\alpha },$$

where β is the normalization constant, and α is the scaling exponent of two interdependent variables of *Y*_1_ and *Y*_2_ [17,30]:

(4) $$\alpha =\frac{dY_{2}/Y_{2}}{dY_{1}/Y_{1}}.$$

That is, the scaling exponent is the rate of change in *Y*_2_ with respect to *Y*_1_. There are three cases for the numerical value of α: (i) α > 1 indicates that increases in *Y*_1_ do not keep pace with the increases in *Y*_2_; (ii) α < 1 indicates that increases in *Y*_2_ do not keep pace with the increases in *Y*_1_; and (iii) α = 1 indicates an isometric relationship between *Y*_1_ and *Y*_2_. Cases (i) and (ii) are referred to as allometric relationships.

Log-transformation of Equation (3) takes the form:

(5) y=γ+αx,

where *y* = log (*Y*_2_), *x* = log (*Y*_1_), and γ = log (β) is the *y*-intercept. Reduced major axis protocols [17] were used to estimate the slope and *y*-intercept.

Given that the numerical value of α might approximate unity, we fixed α to unity and examined the numerical value of β, i.e., an isometric equation (IE):

(6) $$Y_{2}=\beta Y_{1}.$$

We also log-transformed Equation (6) to stabilize the variance of *Y*_2_ and *Y*_1_, i.e.,

(7) y=γ+x,

The bootstrap percentile method [31,32] was used to calculate the 95% confidence intervals (CIs) of the slope (i.e., the scaling exponent, α) and exp (the *y*-intercept) (i.e., the normalization constant, β). The bootstrap percentile method based on 3000 bootstrap replicates was used to test the significance of the difference between any two scaling exponents (or any two normalization constants). If the 95% CI of the differences between the bootstrap replicates of one scaling exponent (or one normalization constant) and those of another scaling exponent (or another normalization constant) includes zero, the difference between the two scaling exponents (or the two normalization constants) is judged to be not significant; if it does not include zero, the difference is judged to be statistically significant.

All analyses were conducted in R (v4.3.1) [28].

## 3. Results

**Figure 2:**
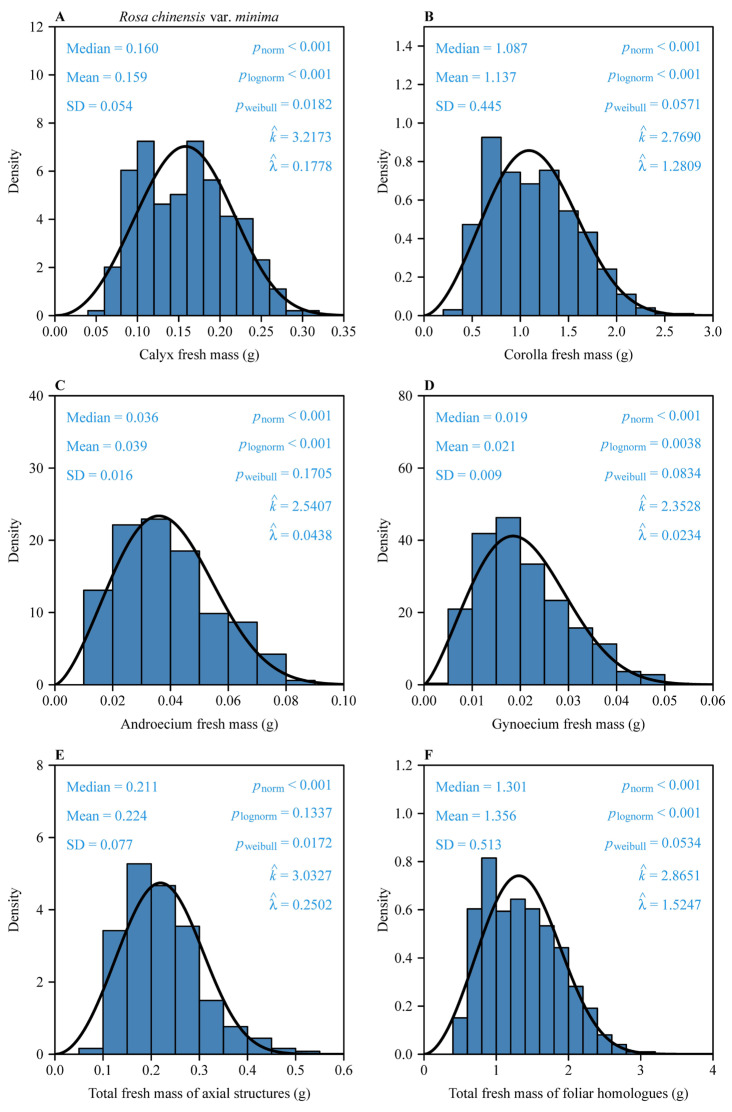
Fresh biomass distributions of six organ type measures: (**A**), calyx; (**B**), corolla; (**C**), androecium; (**D**), gynoecium; (**E**), subtending axial structures [pedicel + receptacle]; and (**F**), foliar homologues [petals + sepals + androecium + gynoecium]) for 497 *Rosa chinensis* var. *minima* flowers. “Mean” and “Median” are the mean and median, respectively; “SD” is the standard error; *p*_norm_ is the probability that the data are consistent with the null hypothesis of a normal distribution; *p*_lognorm_ is the probability that the data are consistent with the null hypothesis of a log-normal distribution; *p*_weibull_ is the probability that the data are consistent with the null hypothesis of the two-parameter Weibull distribution. $\hat{k}$ and $\hat{\lambda }$ are the estimates of the shape and scale parameters in the Weibull probability density function. The solid curves represent the predicted Weibull probability densities.

The fresh mass distributions of all floral components exhibited non-normal and non-log-normal structures, as confirmed by Shapiro–Wilk tests (*p* < 0.05; Figure 2). The two-parameter Weibull distribution provided statistically plausible fits for these organ size measures (corolla, androecium, gynoecium, and foliar homologues with Kolmogorov–Smirnov *p* > 0.05; calyx and subtending axial structures with Kolmogorov–Smirnov *p* > 0.01), with shape parameters *k* ranging between 2.35 and 3.22, indicating consistent right-skewed patterns (Figure 2).

**Figure 3:**
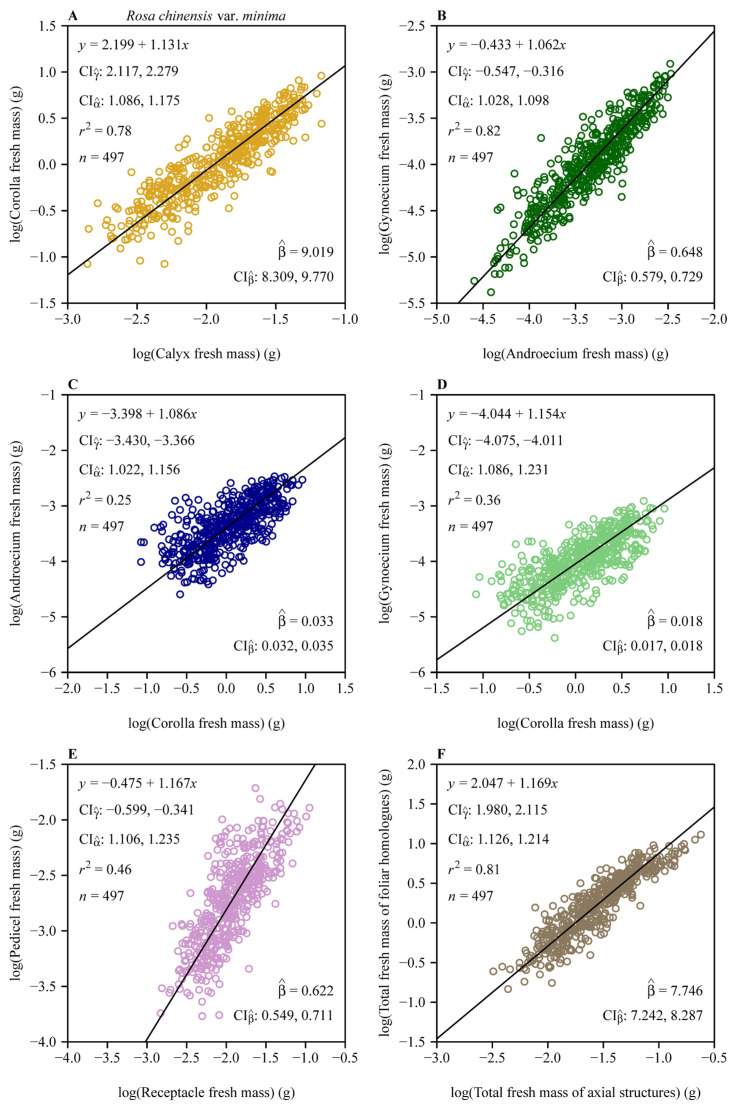
Fitted scaling relationships between any two floral organ size measures on the log–log axis. (**A**) Corolla fresh mass vs. calyx fresh mass; (**B**) gynoecium fresh mass vs. androecium fresh mass; (**C**) androecium fresh mass vs. corolla fresh mass; (**D**) gynoecium fresh mass vs. corolla fresh mass; (**E**) pedicel fresh mass vs. receptacle fresh mass; and (**F**) total fresh mass of foliar homologues vs. total fresh mass of subtending axial structures. The open circles represent the observations; the solids represent the regression lines; ${CI}_{\hat{\gamma }}$ represents the 95% confidence intervals of the estimated *y*-intercept; ${CI}_{\hat{\alpha }}$ represents the 95% confidence interval of the estimated slope; $\hat{\beta }$ is the estimated normalization constant, i.e., exp ($\hat{\gamma }$); ${CI}_{\hat{\beta }}$ represents the 95% confidence intervals of the estimated normalization constant; *r*^2^ is the coefficient of determination; and n is the sample size, i.e., the number of flowers.

Reduced major axis regression revealed pervasive hyperallometric scaling (α > 1) across all pairwise organ comparisons (Figure 3). The androecium versus corolla scaling relationship had α = 1.086 (95% CI: 1.022, 1.156), whereas the gynoecium versus corolla scaling relationship had a steeper slope (α = 1.154, 95% CI: 1.086, 1.231). The subtending axial structures manifested even stronger hyperallometry, with pedicel mass scaling at α = 1.167 (95% CI: 1.106, 1.235) relative to the receptacle. The total of all four foliar homologues biomass scaled at α = 1.169 (95% CI: 1.126, 1.214) relative to the subtending axial structures.

**Figure 4:**
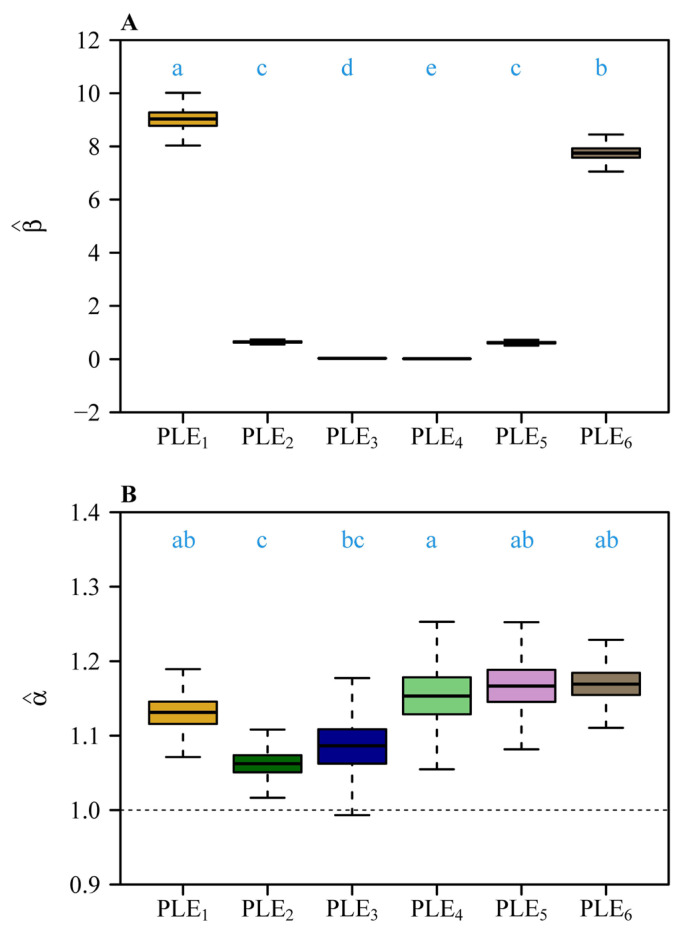
Comparisons of (**A**) the estimated normalization constants among the six scaling relationships (i.e., corolla fresh mass vs. calyx fresh mass, gynoecium fresh mass vs. androecium fresh mass, androecium fresh mass vs. corolla fresh mass, gynoecium fresh mass vs. corolla fresh mass, pedicel fresh mass vs. receptacle fresh, and foliar homologue fresh mass vs. subtending axial structures fresh mass), and (**B**) the estimated scaling exponents among the six scaling relationships. The letters on the top of the whiskers of the boxes signify the significance of the difference in the normalized constants (**A**) or that in the scaling exponents (**B**) between any two scaling relationships; the solid segments in the boxes represent the medians of the normalized constants or the scaling exponents based on 3000 bootstrap replications. “PLE” represents the power-law equation; 1 to 6 correspond to the scaling relationships in Figure 3A–F. The lowercase letters a–e on the top of each box denote the significance of the difference between any two of the six normalization constants (**A**) and that between any two of the six scaling exponents (**B**) based on 3000 bootstrap replicates.

**Figure 5:**
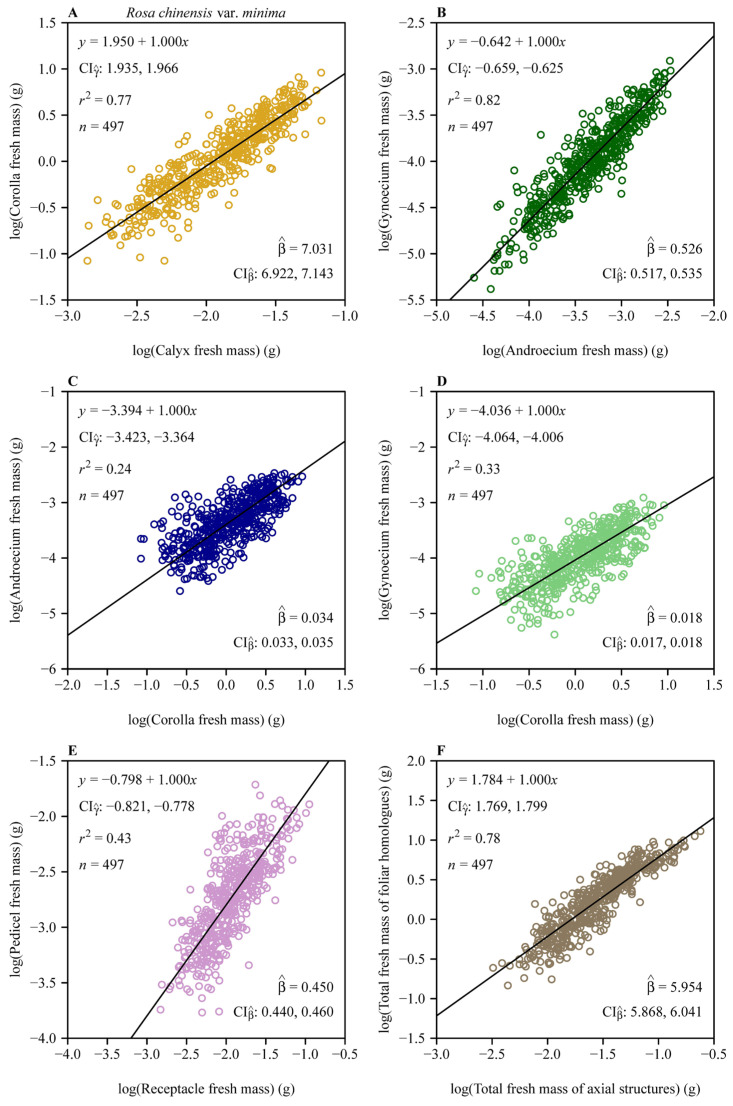
Fitted isometric relationships between any two floral organ size measures on the log–log axis. (**A**) Corolla fresh mass vs. calyx fresh mass; (**B**) gynoecium fresh mass vs. androecium fresh mass; (**C**) androecium fresh mass vs. corolla fresh mass; (**D**) gynoecium fresh mass vs. corolla fresh mass; (**E**) pedicel fresh mass vs. receptacle fresh mass; and (**F**) total fresh mass of foliar homologues vs. total fresh mass of subtending axial structures. The open circles represent the observations; the solids represent the regression lines; ${CI}_{\hat{\gamma }}$ represents the 95% confidence intervals of the estimated *y*-intercept; $\hat{\beta }$ is the estimated normalization constant, i.e., exp ($\hat{\gamma }$); ${CI}_{\hat{\beta }}$ represents the 95% confidence intervals of the estimated normalization constant; *r*^2^ is the coefficient of determination; and *n* is the sample size, i.e., the number of flowers.

**Figure 6:**
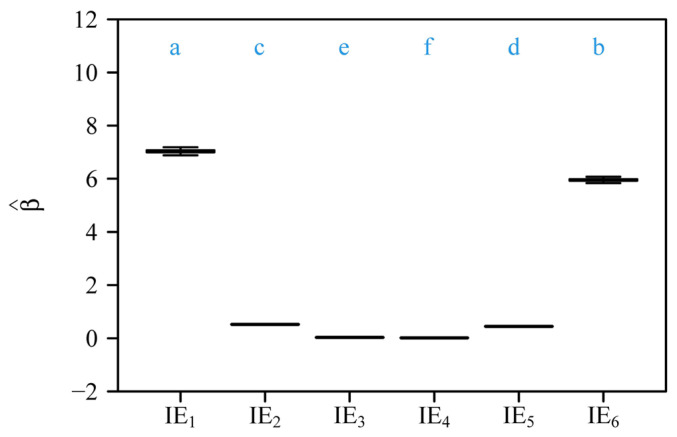
Comparisons of the estimated normalization constants among the six isometric relationships (i.e., corolla fresh mass vs. calyx fresh mass, gynoecium fresh mass vs. androecium fresh mass, androecium fresh mass vs. corolla fresh mass, gynoecium fresh mass vs. corolla fresh mass, pedicel fresh mass vs. receptacle fresh, and foliar homologue fresh mass vs. subtending axial structures fresh mass). The lowercase letters a–f on the top of each box denote the significance of the difference between any two of the six normalization constants based on the 3000 bootstrap replicates; the solid segments in the boxes represent the medians of the normalization constants based on 3000 bootstrap replications. “IE” represents the isometric equation; 1 to 6 correspond to the scaling relationships in Figure 5A–F.

Bootstrap analyses of the six scaling relationships (Figure 4A,B) demonstrated significant differences in both normalization constants (β) and scaling exponents (α) across organ pairs. Pairwise comparisons based on 3000 bootstrap replicates revealed non-overlapping 95% confidence intervals (CIs) for normalization constants (Figure 4A), with distinct alphabetical labels (a, b, c, etc.) indicating statistically significant differences. Similarly, scaling exponents (Figure 4B) differed significantly, as shown by distinct alphabetical labels. The steepest slopes were observed for foliar homologues vs. subtending axial structures (α = 1.169) and pedicel vs. receptacle (α = 1.167), whereas the androecium vs. gynoecium exhibited a comparatively numerically smaller exponent (α = 1.062) (Figure 3 and Figure 4B). Forced isometric models (α = 1) resulted in slightly reduced goodness of fit, as evidenced by lower *r*^2^ values relative to allometric scaling relationships (Figure 5 vs. Figure 3). However, the order of the estimated normalization constants under the isometric hypothesis remained numerically close to those obtained based on the allometric hypothesis, as indicated by the approximate order of alphabetical labels for most pairs (Figure 6 vs. Figure 4A). This partial alignment was interpreted to indicate proportional allocation trends coexist with dominant hyperallometric scaling.

These scaling relationships collectively highlighted differential allocation priorities among floral and subtending modules. The combined biomass of foliar homologues scaled disproportionately against the combined biomass of the subtending structures, indicating a prioritized resource allocation to reproductive and display organs. These patterns are consistent with functional hypotheses linking floral enlargement to enhanced pollinator attraction while maintaining female reproductive capacity through proportional adjustments in organ investment.

## 4. Discussion

The intricate allocation of biomass among floral organs reflects a complex interplay of developmental constraints, ecological pressures, and evolutionary trade-offs. Our study, focusing on *Rosa chinensis* var. *minima*, reveals systematic hyperallometric scaling (α > 1) across all floral and subtending components, with reproductive organs (androecium and gynoecium) exhibiting significant scaling relationships with the corolla. These findings challenge traditional assumptions about proportional resource partitioning in perfect flowers and underscore the need to untangle the mechanisms driving such patterns. The right-skewed Weibull distributions of fresh floral biomasses further highlight the role of biological variability and environmental factors in shaping floral architecture. To contextualize these results, we explore three dimensions: (i) the ecological implications of Weibull-distributed floral biomasses and their deviation from non-reproductive organs, (ii) the adaptive significance of male-biased hyperallometry in hermaphroditic flowers, and (iii) the plasticity of floral allocation strategies under climatic and environmental gradients. By integrating empirical data with theoretical frameworks, this discussion aims to advance our understanding of floral evolution while identifying critical gaps for future research.

### 4.1. Application of the Weibull Distribution and Ecological Implications of Biomass Allocation Patterns

The effectiveness of the two-parameter Weibull distribution in modeling floral organ biomasses (*k* ranging between 2.35 and 3.22) highlights the utility of this protocol in capturing biological variability inherent in conspecific size distributions. Right-skewed distributions, characterized by a long tail toward larger biomasses, likely arise from developmental noise—minor stochastic fluctuations in resource uptake or cellular differentiation, and to environmental factors attending development, such as uneven light exposure, nutrient availability, and mechanical perturbation within monoculture plantings [17,33,34]. The Weibull scale parameter (λ) and derived mean masses reveal significant disparities among organ frequency distributions. For example, the corolla biomass of *R. chinensis* var. *minima* has a mean mass of 1.14 g (Weibull: $\hat{k}$ = 2.77, $\hat{\lambda }$ = 1.28), which is 54-fold higher than that of the gynoecium (0.021 g; $\hat{k}$ = 2.35, $\hat{\lambda }$ = 0.023). This disproportionate allocation aligns with pollinator-mediated selection for larger petals to enhance visibility, whereas minimal investment in the gynoecium ensures efficient resource use [10]. However, the calyx and subtending axial structures deviated significantly from Weibull expectations (*p* < 0.05), which we interpret as a functional decoupling from core reproductive modules. The calyx, initially critical for protecting buds from herbivory and desiccation, transitions post-anthesis to a vestigial role in many Rosaceae species, often persisting as a structural remnant with limited direct reproductive contribution [14]. This ontogenetic shift aligns with the “functional modularity” hypothesis, where floral organs evolve semi-independently under divergent selective pressures. For example, although petals and stamens are often under strong selection for size and symmetry, sepals and pedicles may prioritize their protective and mechanical roles—traits that are less dependent on absolute mass. Experimental manipulations, such as calyx removal or artificial enlargement, could test the possible secondary roles in pollinator attraction or seed dispersal efficiency. Ecologically, the Weibull distribution function’s failure to fit the biomasses of the calyx and subtending axial structures highlights the limitations of assuming a universal or canonical distribution in plant morphology. Future studies should compare alternative models (e.g., gamma and log-logistic) to identify taxon-specific patterns, particularly in non-reproductive structures. Additionally, the skewness parameter (*k*) offers a quantitative metric for comparing developmental stability across populations or species, with lower *k* values indicating greater environmental sensitivity—a potential biomarker for assessing floral resilience to climate change.

### 4.2. Biological Implications of Observed Scaling Relationships

We interpret the pervasive hyperallometry (i.e., scaling exponents numerically exceeding unity, α > 1) observed in *R. chinensis* var. *minima* to reflect a male-biased allocation strategy, where disproportionate investment in larger floral structures enhances male reproductive success (pollen production and export) while maintaining female function (ovule production and successful fertilization). This pattern aligns with classic themes in floral biology, particularly the widespread observation in dioecious and gynodioecious species where male flowers or male-phase organs exhibit larger perianths and greater symmetry to optimize pollen export efficiency [35,36,37]. For example, in dioecious plants, staminate (“male”) flowers often evolve larger petals or inflorescences to attract pollinators and enhance pollen dispersal, that is, a strategy driven by sexual selection for male competition [36]. Similarly, in gynodioecious systems, hermaphroditic (male-fertile) flowers frequently display larger corollas than carpellate (“female”) flowers, reflecting resource reallocation toward pollinator attraction [37]. In the hermaphroditic flowers of *R. chinensis* var. *minima*, the hyperallometric scaling of the corolla and androecium (α > 1) mirrors this male-biased differentiation, suggesting a conserved evolutionary feature: floral enlargement prioritizes male function under pollinator-mediated selection, irrespective of the sexual system. Corolla enlargement ($\hat{\alpha }$ = 1.131) improves pollinator visibility and handling efficiency, as demonstrated in *Ipomopsis aggregata*, where wider corollas correlate with higher hummingbird visitation rates [10]. The steeper scaling of the androecium ($\hat{\alpha }$ = 1.086) and gynoecium ($\hat{\alpha }$ = 1.154) biomasses may ensure synchronized pollen and ovule maturation, critical for avoiding selfing in protandrous flowers [6]. This coordination may provide a developmental “safety net”, wherein female allocation accelerates only after male investment saturates pollinator attraction thresholds. In contrast, the scaling of the calyx deviates from reproductive allometry trends and may indicate its functional redundancy post-flowering. In many *Rosa* species, sepals persist as remnants rather than functional modules, perhaps reflecting an evolutionary trade-off between ancestral protective roles and derived aesthetic functions in horticultural lineages [14]. This divergence illustrates how developmental pathways can be co-opted for novel purposes without disrupting core reproductive modules. For example, it has been suggested that gene regulatory networks regulating sepal growth may have become disassociated from those governing petals and stamens, allowing independent evolutionary trajectories [38].

The hyperallometric prioritization of reproductive organs aligns with the “male function hypothesis”, which posits that pollen export benefits more from floral enlargement than ovule fertilization [19]. This theoretical framework is further supported by historical observations in rose breeding, where artificial selection for ornamental traits (e.g., petal size) indirectly influences reproductive allocation strategies [21]. Specifically, in *R. chinensis* var. *minima*, the hyperallometric investment in corolla and androecium may reflect both pollinator-mediated selection and developmental constraints arising from horticultural practices, such as the co-option of petal expansion pathways at the expense of sepal development. The strong scaling of gynoecium biomass ($\hat{\alpha }$ = 1.154) is consistent with this hypothesis and may indicate that female investment in *R. chinensis* var. *minima* is co-optimized with male allocation to balance reproductive efficiency, challenging the notion of residual female allocation [19]. Future experiments manipulating floral size ranges (e.g., through gibberellin treatments) and quantifying pollen deposition efficiency and seed viability could explicitly test whether female reproductive success is constrained by male-biased allocation in larger flowers.

Beyond evolutionary implications, our findings hold practical relevance for *Rosa* breeding programs. The hyperallometric scaling between reproductive organs and the corolla (α > 1) provides a quantitative framework to predict how artificial selection for ornamental traits—such as double flowers—alters resource allocation. In double-flowered roses, homeotic mutations (e.g., *ROSEA*1 and *ROSEA*2 MADS-box genes) transform stamens and carpels into petaloid structures, thereby disrupting natural scaling patterns [23,39]. Our study shows that wild-type flowers maintain reproductive competence through disproportionate investment in stamens and carpels relative to petals. Conversely, in highly double cultivars, the conversion of reproductive organs into petals likely exacerbates resource competition, potentially explaining reduced fertility without manual pollination [21]. By quantifying scaling exponents in monomorphic flowers like *R. chinensis* var. *minima*, breeders could identify genotypes where petal expansion minimally compromises stamen/carpel biomass, optimizing both aesthetic and reproductive outcomes. For example, selecting for moderate α values might balance doubleness with fertility. Thus, integrating allometric principles into phenotypic screening offers a pathway to develop high-yield ornamental roses resilient to pollination deficits.

The normalized constant β (under the isometric assumption, i.e., α = 1) provides additional insights into baseline proportional allocations among floral organs in *R. chinensis var. minima.* For example, the corolla vs. calyx isometric relationship (Figure 5A) yields $\hat{\beta }$ = 7.031 (95% CI: 6.922, 7.143), indicating that corolla fresh biomass is approximately 7-fold larger than calyx biomass. This aligns with the ecological dominance of petals in pollinator attraction, whereas sepals primarily serve transient protective roles during bud development [10,14]. Similarly, the androecium vs. corolla isometric relationship (Figure 5C; $\hat{\beta }$ = 0.034 with its 95% CI: 0.033, 0.035) highlights minimal stamen investment relative to corolla biomass, which is consistent with the “male function hypothesis”, where indirect traits like petal size enhance pollen export efficiency more than direct stamen biomass [19]. In contrast, the gynoecium vs. androecium isometric relationship (Figure 5B; $\hat{\beta }$ = 0.526 with its 95% CI: 0.517, 0.535) indicates that carpel biomass constitutes approximately 53% of stamen biomass assuming an isometric scaling relationship. We interpret this trend to reflect a compensatory female allocation pattern that improves ovule fertilization despite a male-biased scaling [9]. The second steepest proportionality is seen in the foliar homologue biomass vs. subtending axial structures biomass relationship (Figure 5F; $\hat{\beta }$ = 5.954 with its 95% CI: 5.868, 6.041), where reproductive and display organs outweigh the biomass of supportive structures by nearly sixfold, which can be interpreted to reflect the prioritization of pollinator-mediated traits over mechanical functions [3]. The pedicel vs. receptacle biomass scaling relationship (Figure 5E; $\hat{\beta }$ = 0.450 with its 95% CI: 0.440, 0.460) further shows that pedicel biomass is approximately 45% that of the receptacle biomass, aligning with its dual role in nutrient transport and structural support. These estimated β values under the assumption of isometry (α = 1) reaffirm a hierarchical allocation strategy, mirroring functional prioritization under pollinator-mediated selection. Deviations from isometry (α > 1 in natural scaling; Figure 3) amplify these proportions, as seen in the steeper gynoecium vs. androecium scaling relationship ($\hat{\alpha }$ = 1.062), which may counteract pollen limitation in larger flowers. By reconciling isometric baselines with allometric dynamics, this study reveals how developmental constraints and ecological pressures jointly drive biomass allocation patterns that enhance reproductive functionality.

### 4.3. Environmental Modulation of Floral Resource Allocation

This study examined floral allocation patterns among plants growing under urban (and presumably nearly homogeneous environmental conditions). In contrast, natural populations face climatic and edaphic stressors that likely amplify allocation trade-offs. Drought, for example, can intensify competition for water between vegetative and reproductive tissues, potentially exacerbating floral mass skewness [17]. Recent evidence indicates that xylem cavitation during water stress preferentially damages flowers in woody species [40], which may drive adaptive reductions in corolla investment to conserve resources for gamete development. Such hydraulic constraints could drive the numerical values of scaling exponents toward isometry (α ≈ 1), as observed in herbaceous species where petals and leaves share similar cavitation thresholds [40]. Conversely, nutrient-rich soils can enhance hyperallometry by relaxing resource constraints, allowing for “luxury investment” in pollinator attraction through petal expansion, a strategy analogous to drought-induced xylem segmentation that sacrifices low-cost floral organs to protect vital tissues [41]. Temperature fluctuations further modulate dichogamy timing, and can also potentially decouple male vs. female investment trajectories. Warming climates can advance pollen release before stigmas become receptive, disrupting the synchronized allocation observed here—a scenario documented for the alpine *Polemonium viscosum*, where earlier snowmelt desynchronizes pollinator activity and floral phenology [15]. Similarly, urban “heat islands” can accelerate floral development, shortening the window for optimal pollen transfer.

Future studies should replicate the analyses presented here and elsewhere across altitudinal or latitudinal gradients to unentangle genetic adaptation from phenotypic plasticity. For example, high-altitude populations of *R. chinensis* var. *minima* may exhibit attenuated hyperallometry due to pollinator scarcity favoring autonomous selfing, a trend that is observed in other montane taxa where floral displays are reduced to conserve resources or ensure reproductive assurance under low-pollinator conditions [42,43,44]. Although Arroyo et al. [42] focused on non-Rosaceae species in the high Andes, their findings highlight compensatory mechanisms in high-elevation plants, such as prolonged stigma receptivity and floral longevity, which may mitigate pollen limitation without relying on showy petals [45]. These adaptations may parallel potential strategies in *R. chinensis* var. *minima* to balance pollinator attraction with reproductive resilience in stressful environments. Such insights carry practical implications for horticulture and conservation, for breeders selecting for ornamental traits in *R. chinensis* var. *minima*. Breeders selecting for larger petals must consider the cascading effects on reproductive organ scaling to avoid destabilizing floral function. Conversely, conservationists might prioritize populations exhibiting flexible allocation strategies to buffer against climate variability. Integrating floral allometry into ecological niche models could improve predictions of plant–pollinator network resilience under global change.

## 5. Conclusions

This study provides an analysis of intra-floral biomass allocation and scaling relationships in *Rosa chinensis* var. *minima*, revealing pervasive hyperallometric patterns (α > 1) across all pairwise organ comparisons. The consistent disproportionate investment in larger floral structures, particularly the significant scaling relationships between reproductive organs (androecium and gynoecium) and the corolla, supports the hypothesis that floral enlargement prioritizes male function (pollen production and export) through enhanced pollinator attraction, while maintaining female reproductive effort via coordinated carpel development. This study also shows that the two-parameter Weibull distribution can effectively capture right-skewed biomass distributions of the three floral organ types (corolla, androecium, and gynoecium), with shape parameters *k* smaller than 3.6, thereby confirming non-Gaussian, biologically realistic variability. These findings underscore the role of developmental integration in shaping floral architecture, as predicted by scaling theory, and highlight miniature roses as a tractable system for investigating resource allocation trade-offs in perfect flowers. The disproportionate scaling of foliar homologues (the petals, sepals, androecium, and gynoecium) against subtending axial structures (the pedicel and receptacle) reflects adaptive compromises under pollinator-mediated selection. By aligning with functional hypotheses linking corolla size to pollinator efficiency, this study bridges the gap between morphological adaptation and reproductive ecology, offering empirical evidence for the male-biased investment predicted by sexual selection theory in hermaphroditic flowers. The methodological framework, combining the two-parameter Weibull distribution function with reduced major axis regression protocols, demonstrates the utility of large-sample morphometric analyses in untangling complex allocation strategies, particularly in horticultural species with monomorphic floral structures. By integrating morphometric and scaling analyses, this work underscores the role of developmental constraints in shaping floral architecture and highlights miniature roses as a model for studying intra-floral resource allocation. Although hyperallometric scaling (α > 1) aligns with theoretical predictions for pollinator-mediated adaptation, whether these patterns represent fitness maxima requires future experiments.

## Data Availability

The fresh mass data of the floral organs are available in online Table S1.

## Supplementary Materials

The following supporting information can be downloaded at https://www.mdpi.com/article/10.3390/plants14152446/s1, Table S1: Fresh mass data of floral organs in *Rosa chinensis* var. *minima*.
